# Development of a Real-Time Quantitative PCR Assay for Direct Detection and Quantification of the Root-Lesion Nematode *Pratylenchus penetrans* in Potato Roots

**DOI:** 10.3390/ijms26167711

**Published:** 2025-08-09

**Authors:** Dinesh Poudel, Guiping Yan

**Affiliations:** Department of Plant Pathology, North Dakota State University, Fargo, ND 58108, USA; dinesh.poudel@ndsu.edu

**Keywords:** qPCR, *Pratylenchus penetrans*, potato, root DNA extracts, root-lesion nematode (RLN), qPCR inhibitors, detection, quantification

## Abstract

The root-lesion nematode, *Pratylenchus penetrans*, is a migratory endoparasite that attacks potato roots, causing necrotic lesions and yield losses of up to 73%. Traditional detection and quantification methods are labor-intensive, time-consuming, and require prior nematode extraction and taxonomic expertise. This study aimed to develop a SYBR Green-based real-time quantitative PCR (qPCR) assay for detecting and quantifying *P. penetrans* directly from potato root DNA extracts. Bovine serum albumin (BSA) was tested to neutralize qPCR inhibitors in root DNA extracts. The assay showed high specificity and sensitivity to *P. penetrans*, detecting 1.56 × 10^−2^ of a single nematode in 0.2 g of roots. A standard curve based on artificial nematode inoculations demonstrated a strong linear relationship between Cq values and log-transformed nematode numbers (*R*^2^ = 0.993) with high amplification efficiency. Assessment using root samples from two greenhouse experiments involving five potato cultivars showed strong correlations (*r* = 0.902 and 0.887) between qPCR estimates and microscopic nematode counts. This study presents a new qPCR assay specifically optimized for direct detection and quantification of *P. penetrans* within potato root tissues, offering enhanced sensitivity and applicability for rapid in planta diagnostics to facilitate effective management strategies.

## 1. Introduction

Root-lesion nematodes (RLNs; *Pratylenchus* spp.) are one of the most widespread and economically destructive groups of plant-parasitic nematodes (PPNs) worldwide. These nematodes are ranked third in terms of global economic importance, after root-knot (*Meloidogyne* spp.) and cyst nematodes (*Heterodera* and *Globodera* spp.) [1]. Over 100 *Pratylenchus* species have been identified, affecting over 400 crop species, including potatoes [2,3]. Of the ten RLN species associated with potatoes, *Pratylenchus penetrans* is considered the most damaging [4,5]. This nematode has a broad host range, infecting over 350 plant species, including numerous cultivated crops and weed species, and has been reported in potato fields across North America, Europe, Australia, and Asia [4,6].

*P. penetrans* is a migratory endoparasite that completely enters the root tissues of its host plant, actively moving throughout the root tissues rather than remaining confined to a single feeding site. All life stages of *P. penetrans*, except eggs and first-stage juveniles (J1), are capable of entering and exiting roots, with primary penetration through the zones of root hair and elongation [3,7]. Once inside, the nematode migrates intracellularly by puncturing and degrading the cell walls through mechanical stylet movements and enzymatic secretions, feeding on cortical cells and causing characteristic brown root lesions, root necrosis, and impaired root function [7,8]. The life cycle of the RLNs from egg to egg can be completed within the roots of the host plants and can overwinter in infected root tissues at any life stage, allowing them to persist through unfavorable conditions [3]. At least half of the RLNs present in the field survive inside dead root residues from previous crops [9]. Additionally, about 50% of the RLN population is found in the soil at the beginning and end of the growing season, while during the main growing period, only 20% remains in the soil, with the majority residing in root tissue [9].

*Pratylenchus penetrans* causes significant economic losses in potato production, with yield reductions ranging from 20% to 73% depending upon cultivar susceptibility, initial soil nematode density, and soil and environmental factors [10,11,12,13,14]. In addition to direct damage, *P. penetrans* interacts synergistically with the fungus *Verticillium dahliae*, exacerbating potato early-dying disease and further reducing yields [15]. This disease complex often originates in the root system, where nematode-induced lesions provide entry points for secondary pathogens. Even low densities (as few as 1–2 *P. penetrans* per gram of soil) can reduce potato yield [11]. Although *P. penetrans* inhabits, feeds, and reproduces within root tissues for most of the growing season, damage thresholds based on nematode counts in roots remain limited. Root-based quantification provides a more accurate assessment of impact by an endo-parasitic nematode, better reflecting actual damage risk and guiding effective management decisions. Various management strategies such as chemical control, host resistance, cultural practices, and biological methods can help reduce *P. penetrans* populations [4,13]. However, these strategies depend on accurate species identification and quantification, as the host range and resistance response of host cultivars can vary depending on nematode species.

RLNs primarily target the root system, parasitizing the roots of plant species they infect [3,4,13]. Detection and quantification of the nematodes inside the host plant roots is critical for effective management to prevent further loss by the nematode. Various extraction techniques such as Baermann’s funnel technique, the Whitehead tray method, and centrifugal floatation methods are currently available to extract RLNs from plant tissues and soil particles for morphological and morphometric identification and quantification [16]. However, these traditional extraction and identification methods are labor-intensive and time-consuming, requiring multiple manual steps such as sample preparation, careful setup and handling of plant and soil materials, and lengthy incubation periods to allow nematodes to migrate or float into collection solutions [16,17,18]. In addition, the manual identification and counting of nematodes under a microscope require taxonomic expertise and time, often resulting in a slow and costly diagnostic process. Furthermore, these conventional approaches are not always reliable, as they may not provide accurate estimates due to incomplete nematode recovery and low extraction efficiency in a sample, leading to underestimates of population densities and incomplete assessments of infestation levels [16,19]. Morphological identification is further complicated by the presence of closely related species, such as *P. penetrans*, *P. scribneri*, *P. neglectus*, and *P. crenatus*, which often coexist in the same potato field [20]. These species exhibit overlapping morphological features, often requiring the use of molecular tools for accurate differentiation [2,4].

Molecular diagnostics assays have become an alternative to traditional morphological methods for the detection and quantification of PPNs, offering several advantages [21]. They provide higher accuracy, can detect multiple nematode species in mixed samples, and significantly reduce the time and labor required for diagnosis. Among the various molecular assays, real-time quantitative polymerase chain reaction (qPCR) is one of the more widely used methods and offers high specificity and sensitivity, enabling the detection and quantification of target nematode species simultaneously by comparing the intensity of the amplified signal to the standard curve developed from known population densities [17,22]. Several qPCR assays have been developed to detect nematodes directly from infected plant tissues for different PPNs, including a root-lesion nematode *P. scribneri* in potato roots [17]. Currently, conventional PCR methods are available for the indirect detection of *P. penetrans* from the soil and root, requiring nematode extraction from soil and plant material prior to DNA isolation [23,24]. Direct detection has also been achieved using conventional PCR using DNA extracted from peels of infected potato tubers [14]. In addition, qPCR assays have also been developed for indirect detection of *P. penetrans* [25,26], as well as for direct detection and quantification using DNA extracted from soil samples [22]. While these approaches represent significant advances, soil-based detection may not accurately reflect the population of nematodes actively feeding within potato roots, as soil DNA can originate from dormant or dead nematodes not currently impacting the crop. Additionally, soil- and root-based DNA samples may differ in terms of co-extracted organisms and chemical composition, which can impact the sensitivity and reliability of detection assays. At present, there is no root-based qPCR assay available for the direct detection and quantification of *P. penetrans* in potato roots, the primary site of infection and feeding. A root-based detection and quantification assay is essential, as it more accurately reflects the active infestation and potential crop damage, allowing for continuous monitoring of nematode levels and effective management interventions. These factors highlight the need for a rapid, accurate, root-based, cost-effective and user-friendly diagnostic tool for direct detection and quantification of *P. penetrans* in potato roots.

Developing qPCR assays for root tissues presents several challenges due to the co-extraction of PCR inhibitors such as polysaccharides, polyphenols, pectin, xylan, and residual ethanol during DNA extraction [27,28]. These compounds can interfere with DNA polymerase activity, reducing amplification efficiency and potentially causing false negatives or inconsistent quantification. While diluting samples can reduce inhibition [29], it also dilutes the target DNA, potentially lowering qPCR sensitivity. Therefore, optimizing qPCR assays to neutralize these inhibitors is critical for achieving high sensitivity. The addition of BSA enhances PCR efficiency by binding to inhibitors, reducing their interference with DNA polymerase and improving PCR inhibitor tolerance [30]. Nevertheless, the complexity of root tissue samples requires careful optimization and validation of both DNA extraction and amplification protocols.

The goal of this study was to develop a SYBR Green-based qPCR assay for the rapid, direct detection and quantification of *P. penetrans* in infected potato root tissues. The specific objective was to develop a species-specific qPCR assay targeting *P. penetrans* in DNA extracts from infected potato roots by assessing the presence of PCR inhibitors in root DNA extracts, evaluating the assay’s specificity and sensitivity, and testing the assay using DNA extracted from infected root samples of different potato cultivars.

## 2. Results

### 2.1. Nematode Species Confirmation as P. penetrans

The species identity of *P. penetrans* extracted from both greenhouse-maintained potato plants and carrot disc cultures was confirmed using the species-specific primers PP5F/PP5R. PCR amplification produced a clear, single band of approximately 520 bp in all tested samples and positive controls, consistent with the expected fragment size for *P. penetrans* [24]. No amplification was observed in the negative controls consisting of *P. dakotaensis*, *P. neglectus*, *P. scribneri*, and Hg51 unnamed RLN (Supplementary Figure S1). These results confirm the species of RLN as *P. penetrans*.

### 2.2. Specificity of the qPCR Assay

**Figure 1:**
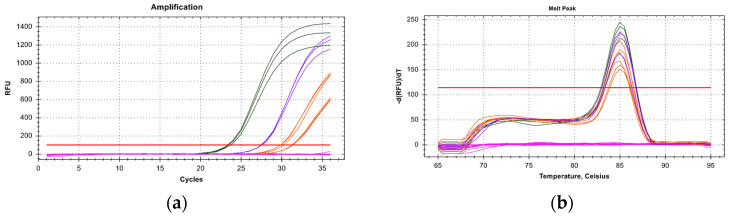
Specificity test of the qPCR assay for *Pratylenchus penetrans*, performed using DNA extracted from 0.2 g of ‘Red Norland’ potato root tissue artificially inoculated with a single nematode of *P. penetrans*, other *Pratylenchus* species, or common nematodes found in Minnesota (MN) and North Dakota (ND). (**a**) Amplification curves using primers Pp-F/Pp-R showing specific amplification of *P. penetrans* DNA (orange curves). No amplification was observed in negative controls and root DNA extracts containing non-target nematode (magenta curves) lacking *P. penetrans* DNA. (**b**) Melting curve profiles showing a single peak at 85 °C for *P. penetrans* amplicons, confirming primer specificity. Positive controls consisted of DNA extracted by the Proteinase K method from either four *P. penetrans* (green curves) or a single *P. penetrans* (purple curves). Negative controls included a non-template control (ddH_2_O instead of DNA template) and DNA from uninfected ‘Red Norland’ roots, both showing no amplification.

The specificity test of the Pp-F/Pp-R primer pair confirmed its ability to specifically detect *P. penetrans* in 0.2 g of potato roots. Amplification was observed only in positive controls and DNA extracts from roots inoculated with *P. penetrans*, while no amplification occurred for non-template controls and the negative controls (DNA extracts from roots artificially inoculated with other *Pratylenchus* species and plant-parasitic nematodes tested) (Table 1). The quantification cycle (Cq) values for the two positive controls used were 23.43 ± 0.17 (DNA extracted using the Proteinase K method with four *P. penetrans* as the DNA template) and 27.41 ± 0.12 (DNA extracted by the same method with one *P. penetrans* as the template). The two DNA samples extracted from 0.2 g of potato roots inoculated with a single *P. penetrans* yielded Cq values of 29.92 ± 0.06 and 31.21 ± 0.22. All other DNA extracts included in the specificity test showed no amplification (Figure 1a). A single melting peak with a melting temperature of 85 °C was observed in the qPCR assay, confirming the amplification of a single, specific amplicon (Figure 1b).

### 2.3. Detection of PCR Inhibitors in Potato Roots and Optimization Using BSA

The qPCR analysis showed that the root samples harvested at 25 days after planting (DAP) (DP2) had the highest average Cq value (21.09 ± 0.15), indicating that they had the most significant PCR inhibition (Table 2). In contrast, the root samples harvested at 20 DAP (DP1), 30 DAP (DP3), and 35 DAP (DP4) exhibited similar Cq values (19.32 ± 0.11, 19.15 ± 0.13, and 19.19 ± 0.09, respectively), which were not significantly different from one another but remained higher than the positive control. The positive control, with the lowest average Cq value (16.92 ± 0.10), demonstrated an effective qPCR performance without inhibitors. These findings confirm the presence of PCR inhibitors in the root DNA extracts and highlight the necessity of optimizing the qPCR assay to mitigate their effects.

The addition of BSA to the qPCR reaction mixture demonstrated its effectiveness in reducing the inhibitory effects of root DNA extracts, as evidenced by decreasing Cq values (Table 3). Without BSA, the average Cq value was 19.69 ± 0.85, which was significantly higher than all BSA-treated reactions. The inclusion of BSA at 0.2 µg/µL reduced the Cq to 18.52 ± 0.57, while further increases in BSA concentration to 0.3 µg/µL and 0.4 µg/µL lowered the Cq values to 18.43 ± 0.52 and 17.72 ± 0.48, respectively. The Cq values for 0.4 µg/µL (17.72 ± 0.48) and 0.5 µg/µL (17.78 ± 0.33) were statistically similar to the positive control (16.92 ± 0.10), which consisted of plasmid DNA and ddH_2_O without root DNA. This indicates that at these higher BSA concentrations, the inhibitory effects of root DNA extracts were effectively neutralized, achieving a performance comparable to the positive control. These results indicate that BSA concentrations of 0.4 µg/µL and 0.5 µg/µL are optimal for mitigating PCR inhibitors in root DNA extracts, enhancing qPCR assay accuracy.

### 2.4. Sensitivity of the qPCR Assay

The detection sensitivity of the qPCR assay was evaluated using two-fold serial dilutions of DNA extracted from 0.2 g of potato roots artificially inoculated with four *P. penetrans*, down to a 1/1024 dilution (equivalent to 3.91 × 10^−3^ nematodes per 0.2 g of roots) (Table 4). The qPCR assay detected DNA at dilutions as low as 1/256 (1.56 × 10^−2^ nematodes per 0.2 g of roots), yielding a Cq value of 34.77 ± 0.07. Amplification was not observed at dilutions of 1/512 or 1/1024. Across the serial dilutions, Cq values ranged from 27.72 ± 0.14 for undiluted DNA to 34.77 ± 0.07 at the detection threshold, with no amplification in non-template and negative controls. Positive controls included DNA extracted using the Proteinase K method with one or four *P. penetrans* added to the reaction mixture, yielding Cq values of 27.30 ± 0.07 and 23.38 ± 0.08, respectively, demonstrating good amplification. The Cq values and the log-transformed starting DNA quantity from the serial dilutions exhibited strong linearity (*R*^2^ = 0.980) and an amplification efficiency of 106.47%, described on the regression equation, y = −3.176x + 28.884.

### 2.5. Development and Validation of Standard Curve

**Figure 2:**
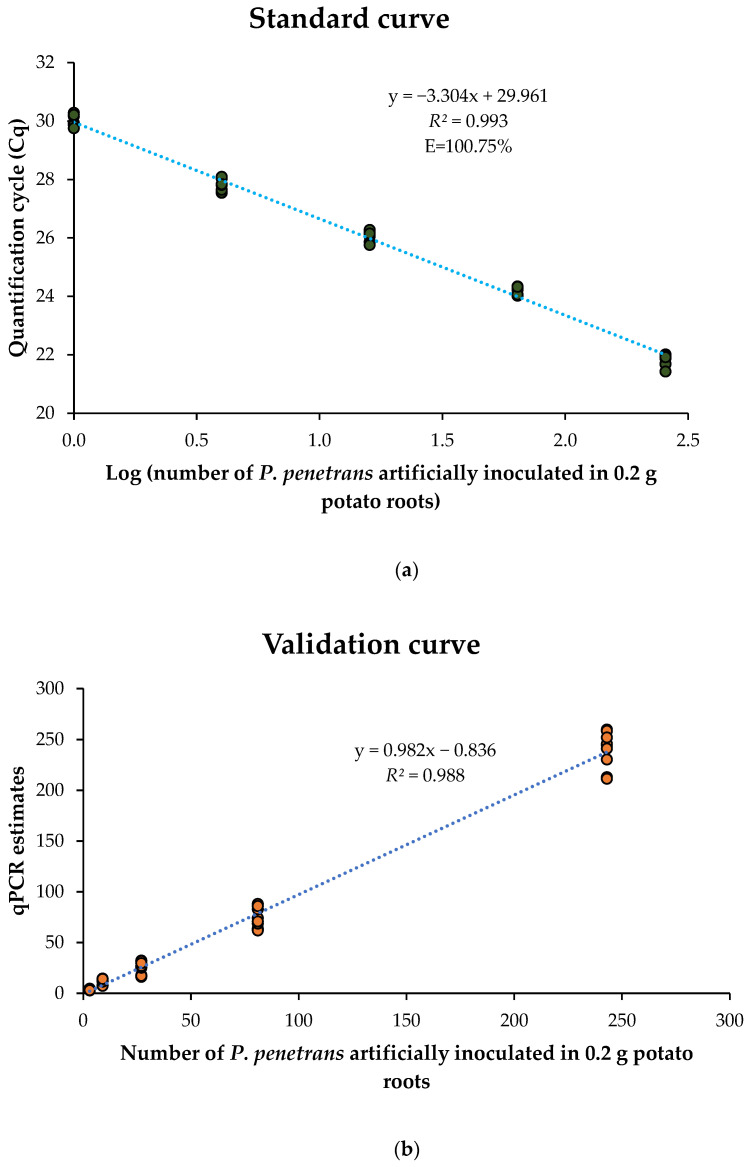
Generation and validation of the standard curve for the qPCR assay for *Pratylenchus penetrans*. (**a**) Standard curve showing the relationship between the quantification cycle (Cq) and the logarithm (log_10_) of *P. penetrans* densities (1, 4, 16, 64, and 256) artificially inoculated in 0.2 g of non-infected ‘Red Norland’ potato roots. Each green dot represents the mean Cq value (*n* = 9) from three biological replicates, with each replicate analyzed in triplicate in the qPCR assay. Amplification efficiency, E, was calculated as E = 10 ^(1/–m)^ − 1, where ‘*m*’ is the slope of the standard curve equation. (**b**) Validation curve obtained by plotting qPCR estimates obtained from the standard curve equation against the actual *P. penetrans* densities (3, 9, 27, 81, and 243). Each orange dot represents the mean Cq value (*n* = 9) from three biological replicates, with each replicate analyzed in triplicate in the qPCR assay.

The standard curve was developed by artificially inoculating different densities (1, 4, 16, 64 and 256) of *P. penetrans* in 0.2 g of potato roots. The relationship between the Cq values and the corresponding log_10_ *P. penetrans* densities was described by the equation y = −3.304 + 29.961 (Figure 2a). The Cq values for nematode densities of 1, 4, 16, 64, and 256 per 0.2 g of roots were 29.99 ± 0.18, 27.79 ± 0.22, 26.08 ± 0.17, 24.20 ± 0.13, and 21.84 ± 0.20, respectively. No amplification was observed in the non-template control which used ddH_2_O instead of template and negative controls which used DNA extracted from uninfected potato roots as the template. A strong inverse linear relationship was observed between Cq values and the log_10_ *P. penetrans* densities (*R*^2^ = 0.993), and the amplification efficiency was calculated to be 100.75%.

To validate the standard curve, DNA extracted from potato roots artificially inoculated with *P. penetrans* densities (3, 9, 27, 81, and 243) not used in the curve development was tested using the qPCR assay. The Cq values ranged from 22.10 ± 0.11 for 243 nematodes to 28.19 ± 0.26 for 3 nematodes per 0.2 g of roots. The qPCR-based estimates of *P. penetrans* densities, derived from the standard curve equation, exhibited a strong positive correlation (*R*^2^ = 0.988) with the actual inoculated *P. penetrans* densities. This relationship was described by the equation y = 0.982x − 0.836 (Figure 2b).

### 2.6. Mean Final Population Density of P. penetrans in Roots of Potato Cultivars

Microscopic counts of *P. penetrans* extracted from potato roots revealed significant differences in final population density among the five cultivars evaluated (*p* < 0.0001) (Table 5). In Trial 1, Red Norland supported the highest root population (5420 ± 403), followed by Caribou Russet (4728 ± 773) and Yukon Gold (3712 ± 342). In contrast, significantly lower densities were observed in Colomba (2101 ± 262) and Modoc (1952 ± 175). A similar trend was observed in Trial 2, where Red Norland again exhibited the highest root population (4023 ± 411), followed by Caribou Russet (3318 ± 412) and Yukon Gold (3287 ± 370). Colomba (1880 ± 291) and Modoc (1709 ± 187) maintained the lowest root populations.

Quantification of *P. penetrans* using the qPCR assay corroborated the microscopic findings, with significant differences observed among cultivars in both trials (*p* < 0.0001) (Table 5). In Trial 1, the highest population was detected in Red Norland (5473 ± 634), followed by Caribou Russet (4558 ± 942) and Yukon Gold (3863 ± 707). Lower populations were observed in Modoc (2276 ± 199) and Colomba (2166 ± 372), which were statistically similar to each other and significantly lower than the other cultivars. In Trial 2, Red Norland again had the highest estimated population (5141 ± 731), followed by Caribou Russet (4091 ± 1381) and Yukon Gold (3725 ± 505). Colomba (2497 ± 609) and Modoc (2087 ± 294) had the lowest nematode populations.

Paired comparisons between microscopic counts and qPCR estimates of *P. penetrans* populations in potato roots revealed no significant differences for any of the cultivars in Trial 1 (Table 5). In Trial 2, however, significant differences were observed for four cultivars: Red Norland (*p* < 0.05), Yukon Gold (*p* < 0.01), Modoc (*p* < 0.01), and Colomba (*p* < 0.05), with qPCR consistently estimating higher nematode densities than microscopic counts (Table 5). Caribou Russet showed no significant differences between methods in either trial.

### 2.7. Assessment of qPCR Assay for in Planta Detection of P. penetrans

**Figure 3:**
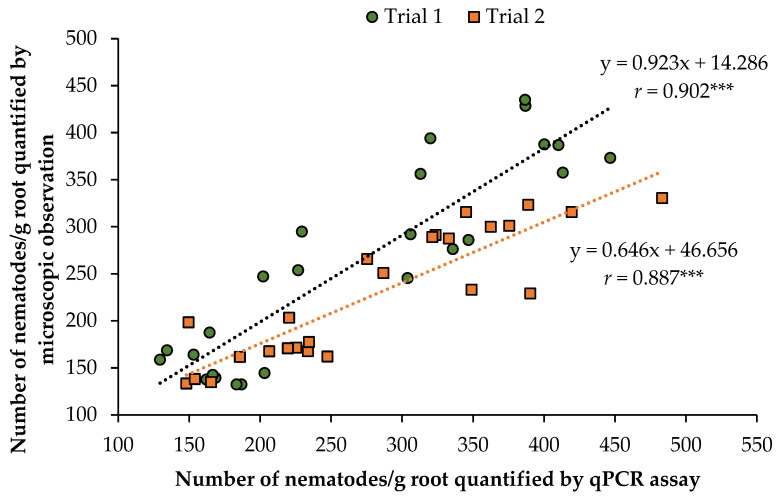
Correlation analysis between the *Pratylenchus penetrans* densities per grams of root obtained through Whitehead tray extraction followed by microscopic counting and those quantified using the qPCR assay for five potato cultivars with five replications in the greenhouse. Each data point represents one of 25 root samples per trial (5 cultivars × 5 greenhouse replicates). The green circle represents samples from Trial 1, where the number of *P. penetrans* quantified by qPCR assay is the mean of three independent biological replicates and qPCR ran in three technical replicates (*n* = 9), and the corresponding value for microscopic counts is the mean of three independent Whitehead tray extractions (*n* = 3). Orange squares represent samples from Trial 2, where qPCR quantification was performed as in Trial 1 (*n* = 9), and the corresponding microscopic count is based on a single Whitehead tray extraction. Regression lines and correlation coefficients are displayed for each trial to illustrate the relationship between two quantification methods. *r* indicates the Pearson correlation coefficient. *** Significant at *p* < 0.0001.

The accuracy of the developed qPCR assay was evaluated by comparing qPCR-based quantification with microscopic counts from Whitehead tray extractions of infected potato roots. Root samples from five potato cultivars exhibiting varying levels of nematode reproduction were analyzed in two independent greenhouse trials (Table 5). Across both trials, the average number of nematodes per gram of root was consistent between the two methods for all cultivars. In Trial 1, Red Norland exhibited the highest nematode densities (393 ± 47 by qPCR; 389 ± 29 by Whitehead tray extraction and microscopic counting), followed by Caribou Russet (337 ± 71; 350 ± 61), Yukon Gold (275 ± 57; 263 ± 20), Modoc (167 ± 11; 143 ± 12), and Colomba (164 ± 32; 158 ± 21). A strong and statistically significant positive correlation (*r* = 0.902, *p* < 0.0001) was observed between the two methods, described by the equation y = 0.923x + 14.286 (Figure 3), confirming the assay’s high accuracy. Similarly, in Trial 2, similar trends were observed: Red Norland (375 ± 62; 293 ± 37), Yukon Gold (308 ± 61; 271 ± 46), Caribou Russet (322 ± 111; 262 ± 49), Colomba (212 ± 39; 160 ± 16), and Modoc (192 ± 34; 156 ± 19). Strong and statistically significant correlation (*r* = 0.887, *p* < 0.0001) was observed, and described by the regression equation y = 0.646x + 46.656 (Figure 3).

## 3. Discussion

In this study, we developed a qPCR assay for the detection and quantification of *P. penetrans* directly from potato roots. This assay offers a rapid, sensitive, and specific method for detecting *P. penetrans* without the need for prior nematode extraction. The addition of BSA to the qPCR reaction mix neutralized the effects of PCR inhibitors present in the root DNA extracts, improving amplification efficiency. The assay demonstrated high specificity, detecting *P. penetrans* without cross-reacting with closely related *Pratylenchus* species. Furthermore, it showed strong sensitivity, detecting DNA equivalent to less than a single nematode, and it was tested on greenhouse-grown infected root samples, supporting its practical application in nematode diagnostics.

Several qPCR assays for detecting RLNs have been developed for the detection of root-lesion nematodes (RLNs), including *P. scribneri*, *P. neglectus*, *P. penetrans*, *P. thornei*, and other species which are commonly encountered in potato production systems [22,32,34,35]. However, the majority of these assays are designed to work with soil samples, and studies focusing on the direct detection and quantification of RLNs from infected plant tissues are limited. A qPCR assay has been developed for detection and quantification of *P. scribneri* in potato roots, demonstrating the feasibility of root-based molecular diagnostics [17]. A conventional PCR has also been developed for direct detection of *P. penetrans* from potato tubers [14]; however, it does not allow quantification of nematode densities. *P. penetrans*, due to its endo-parasitic nature, resides predominantly within root tissues for most of the growing season [9], making root-based detection particularly relevant for accurate diagnosis and management. Although both TaqMan [26] and SYBR Green [22,25] chemistries have been used to detect *P. penetrans,* either indirectly (requiring prior nematode extraction) or directly from soil, there have been no reports of direct detection and quantification from infected roots, which represent the primary site of nematode colonization. In this study, a SYBR Green-based qPCR assay was developed for the detection and quantification of *P. penetrans* directly from infected potato roots. Real-time qPCR is widely used for nematode detection due to its reliability, sensitivity, rapid turnaround, cost-effectiveness, and high-throughput capability [22,27]. Compared to TaqMan, SYBR Green-based qPCR is simpler and more cost-effective, as it does not require labeled probes, and can achieve comparable results when optimized appropriately [36]. By enabling direct detection from potato roots, the qPCR assay developed in this study addresses a critical gap in *P. penetrans* diagnostics and provides a valuable tool for improving nematode management in potato production systems.

The accurate identification and quantification of *P. penetrans* have become increasingly important due to its impacts on different crops. For species-specific detection of *P. penetrans*, we used the D2-D3 28S rDNA primer pair developed by Baidoo et al. [22], which amplifies a 111 bp product specific to *P. penetrans* from soil DNA. While Baidoo et al. [22] initially evaluated specificity across eight isolates of *P. penetrans*, 12 isolates of *Pratylenchus* spp., and 19 isolates of other plant-parasitic nematodes, we re-evaluated specificity in our study. This re-examination was necessitated by differences in sample preparation and potential interference from co-extracted organisms, including plant root DNA present in our extracts. To strengthen this analysis, we included two newly reported RLN species from North Dakota (ND), *P. dakotaensis* [31] and the Hg51 unnamed RLN [33]. Melt curve analysis revealed a single peak, confirming specific amplification of *P. penetrans* and no cross-reactivity with non-target species. The relative stability of the D2-D3 region across *Pratylenchus* species, compared to the polymorphic nature of ITS sequences, further supports its suitability for designing and selecting species-specific primers for *P. penetrans* [22,23].

The presence of qPCR inhibitors such as polysaccharides, polyphenols, pectin, humic acids, xylan, and residual ethanol during DNA extraction can interfere with accurate qPCR results, especially when working with complex plant tissues [28]. In this study, DNA extracts from potato roots at different DAP showed varying levels of inhibition based on Cq values. The DNA extract from 25 DAP (DP2) had the highest Cq value (21.09 ± 0.15), indicating the most inhibition, while extracts from 20, 30, and 35 DAP (DP1, DP3, DP4) had lower Cq values (~19), though they were still higher than the positive control (16.92 ± 0.10). This suggests that specific inhibitors and their concentrations in root samples can vary depending on the plant growth stage, necessitating further investigations into the biochemical composition of the extracts over time to determine the specific inhibitors present. The addition of BSA in the qPCR reaction mix neutralized the inhibitory effects observed in the root DNA extracts. The reduction in Cq values correlated positively with increasing concentrations of BSA, supporting findings from previous research that showed that the addition of BSA enhances the qPCR performance by binding inhibitory compounds and preventing their interaction with DNA polymerase [30,37]. In our assay, the addition of 0.4 µg/µL of BSA to the qPCR reaction mix effectively lowered Cq values to levels comparable with the positive control, confirming its efficacy in neutralizing qPCR inhibitors present in potato roots. Similar improvements in PCR performance with addition of 0.2–0.4 µg/µL BSA have been reported in previous studies when working with water samples and soil RNA and DNA extracts [38,39]. Optimal qPCR performance depends on reducing inhibition while maintaining specificity, sensitivity and accuracy. Diluting DNA extracts can reduce inhibition; however, it also decreases the target DNA concentration [29]. This compromises assay sensitivity, particularly in samples with low DNA targets. The results of this study highlight the importance of considering sample-related inhibitors during assay design. The use of BSA proved effective in improving amplification from inhibitor-rich plant samples, supporting its inclusion in standardized protocols to enhance the reliability of qPCR diagnostics. However, qPCR inhibition may be more pronounced at low template concentrations [30,40], where higher BSA levels or inhibitor-tolerant reagents might be necessary. Further testing of BSA titration under low-template conditions may help ensure a robust performance at different template concentrations.

The evaluation of the detection sensitivity of the developed qPCR assay demonstrated a lower detection limit at the 1/256th dilution in a serial dilution series initiated with four *P. penetrans* individuals. This corresponds to an estimated concentration of approximately 1.56 × 10^−2^ *P. penetrans* per 0.2 g of potato root tissue. Comparable sensitivity levels have been reported in previous studies using real-time qPCR for other *Pratylenchus* species. For instance, Arora and Yan [17] developed a qPCR assay capable of detecting as little as 7.8 × 10^−3^ *P. scribneri* per 0.2 g of potato roots. Similarly, comparable sensitivity has also been achieved in soil-based qPCR assays, with previous studies demonstrating the ability to detect a single individual of *P. penetrans*, *P. thornei*, or *P. neglectus* in 1 g of soil using DNA extracted directly from soil samples [22,32,34]. While some studies demonstrate the potential of qPCR for detecting low populations of nematodes in soil, reports on detection directly from host root tissues remain limited, and evaluations of assay sensitivity in root tissue are even rarer. Currently, established economic thresholds for *P. penetrans* in root tissues are lacking. However, several studies have defined thresholds in soil. It has been reported that crop damage can occur at soil populations as low as 1–2 *P. penetrans* per gram, depending on the potato cultivar and environmental conditions [11,12]. These thresholds are influenced by various factors, including crop type, nematode distribution, soil properties, climatic conditions, and the potential for synergistic interactions with other pathogens [13,22]. Given that nematode infestation in roots may be more closely linked to immediate crop damage than soil populations, the ability of the qPCR assay developed in this study to detect as little as 1.56 × 10^−2^ nematodes per 0.2 g of root tissue demonstrates its potential utility as a sensitive diagnostic tool for detection.

The qPCR assay enables simultaneous detection and quantification of the nematodes by comparing the intensity of amplified signal to the standard curve developed from known population densities. The standard curve in this study was generated by artificially inoculating known nematode individuals, comprising a mixed population of juveniles and adults of *P. penetrans* in 0.2 g uninfected potato roots. The development of standard curves for real-time qPCR applications is a well-established practice, with methodologies including the initial serial dilution of DNA obtained from the known initial number of nematodes [26] or the incorporation of varying nematode numbers into soil or root samples [17,22]. The latter approach is often regarded as more representative of natural field conditions, thus making the results more applicable [22]. In this study, the standard curve demonstrated an amplification efficiency of 100.75%, which falls within the ideal range of 90–110% considered acceptable for reliable qPCR quantification [41]. Comparable amplification efficiencies have been reported in previous studies, such as 104% in the qPCR assay developed by Mokrini et al. [26] for *P. penetrans*, and 103% by Arora and Yan [17] in their assay targeting *P. scribneri* directly from potato roots. Furthermore, the standard curve generated in this study was validated, showing a strong correlation (*R*^2^ = 0.988) between the qPCR-derived estimates and the actual nematode densities used for validation, thus asserting the efficacy and accuracy of the assay for quantitative detection of *P. penetrans* in potato root tissue.

The population distribution of RLNs in soil and roots is highly variable and is influenced by several factors, including the availability of food, plant growth stage, root system development, and environmental conditions [5,31]. RLNs are migratory endoparasites that can move freely between the root and the soil. Above 50% of the RLNs reside within the host root tissue during periods of active plant growth and when host roots are abundant [31]. MacGuidwin [9] also reported that the *P. scribneri* populations recovered from roots were significantly larger in samples collected from early to late in the growing season, compared to those taken at the very beginning or end of the season. The damage caused by endo-parasitic nematodes is more closely related to the number of nematodes that penetrated and are present within the roots [42]. The cultivars supporting higher RLN populations in their roots also tend to have higher final nematode densities and reproductive factors, reflecting the nematode’s ability to multiply effectively when conditions within the roots are favorable [5,31]. In this study, we assessed the final population densities of *P. penetrans* in the roots of five potato cultivars commonly grown in ND and MN, using a nematode isolate collected from MN. Previous studies have shown that nematode reproduction is influenced by both potato genotype and nematode isolate [43]. Significant differences in nematode populations were observed among the cultivars. ‘Red Norland’ which was used as a susceptible host in previous study [5] maintained the highest population in roots. In contrast, ‘Modoc’ and ‘Colomba’ maintained comparatively lower nematode populations, although both were still suitable hosts for *P. penetrans*. Overall, Colomba and Modoc exhibited lower susceptibility to *P. penetrans* compared to Red Norland, Caribou Russet, and Yukon Gold. The infected roots from the greenhouse experiments were subsequently used to validate the developed qPCR assay.

In this study, the qPCR assay was evaluated in two independent greenhouse experiments using five potato cultivars. To establish a baseline for quantifying and comparing nematode populations, population densities were assessed using both the qPCR assay and conventional microscopy following the Whitehead tray extraction method. A strong and significant positive correlation was observed between the two methods (*r* = 0.902 and 0.887 in Trials 1 and 2, respectively), supporting the reliability of the qPCR assay as an alternative to conventional microscopy for nematode detection and quantification. Correlations were even stronger in artificially inoculated root samples, where a known number of *P. penetrans* were added to 0.2 g of uninfected root tissue. In these controlled samples, a near-perfect correlation (*R*^2^ = 0.988) was observed between the actual number of nematodes and qPCR estimates, indicating high assay accuracy under ideal conditions. This pattern of higher correlation in controlled inoculations compared to more variable greenhouse or field samples has also been reported in previous studies [17,22]. No significant differences were observed between the two methods across the five potato cultivars in the first trial. However, in the second trial, the qPCR assay slightly overestimated *P. penetrans* numbers compared to microscopic counts in most of the potato cultivars tested, consistent with earlier findings [22]. This discrepancy may have been the result of incomplete nematode recovery using Whitehead tray extractions and the limitations of the microscopy method when it comes to detection and quantification based on the morphology of vermiform stages. Additionally, in the second trial, only one extraction was performed per greenhouse replicate, which may also have contributed to the observed variation.

Quantitative PCR results can be influenced by several factors that affect the accuracy of nematode quantification. For example, qPCR detects total DNA from both living and dead nematodes, as well as from eggs, which may lead to an overestimation of viable nematode populations compared to microscopy, which typically identifies only live and motile individuals [17,22,44,45,46]. However, in this study, dead nematodes were unlikely to be present because samples were collected while the plants were healthy; therefore, any overestimation likely resulted from the detection of eggs rather than nonviable nematodes. The consistency of qPCR quantification also depends on the efficiency of DNA extraction and technical variation between runs. To reduce quantification bias arising from technical variability and differences in PCR efficiency, we initially developed a standard curve using potato roots inoculated with known densities of *P. penetrans*, which was then tested using infected root samples of different potato cultivars. While the complete set of standard samples was not included in every qPCR run, two representative standard samples (1 and 256 *P. penetrans* per 0.2 g roots) were consistently run alongside non-template and negative controls to monitor assay performance. We used a commercial DNA extraction kit in this study, but PCR inhibitors present in plant tissues, together with the challenge of lysing nematodes with a tough cuticle, can still reduce DNA yield or affect amplification efficiency [28,47]. To minimize inhibitor effects, BSA was added to the qPCR reactions. Alternatively, recent advances in inhibitor-tolerant qPCR reagents have been shown to enhance performance in the presence of inhibitors [48,49]. Such reagents could eliminate the need for additives like BSA and reduce optimization requirements across different potato cultivars, thereby streamlining qPCR workflows for future plant diagnostics. Despite these inherent challenges, the strong correlation between qPCR estimates and microscopic counts observed in this study indicates that the qPCR assay provides a robust, sensitive, and high-throughput tool for detecting and quantifying *P. penetrans* in planta.

Real-time qPCR has been effectively utilized in resistance phenotyping for other plant-parasitic nematodes. For instance, Ruiz et al. [50] developed a qPCR assay to quantify *Tylenchulus semipenetrans* in citrus roots, facilitating rapid screening of resistant rootstock. Building on these successful applications, the present study lays a strong foundation for employing a qPCR assay to screen potato cultivars for resistance to *P. penetrans*. Further validation using a diverse set of resistant and susceptible potato cultivars is warranted to confirm its effectiveness. Additionally, evaluating the assay across multiple plant growth stages may enhance its ability to facilitate early and high-throughput screening in resistance breeding programs. In addition, with further testing, this assay holds promise as a tool for establishing damage thresholds in potato roots and supporting informed crop management decisions.

## 4. Materials and Methods

### 4.1. Nematode Species Confirmation

Populations of *P. penetrans,* initially collected from Becker County, MN, were maintained under greenhouse conditions on the susceptible potato cultivar ‘Red Norland’ and on carrot discs at the North Dakota State University nematology laboratory. Nematodes were extracted from both greenhouse samples using the sugar centrifugal flotation technique [51] and from six-month-old *P. penetrans* carrot disc cultures incubated at 22 °C. Individual RLNs were picked manually based on morphological characteristics using a dental pick from both samples, and DNA was extracted using the Proteinase K method [22]. Previously confirmed *P. penetrans* DNA samples served as positive controls and DNA extracted from other *Pratylenchus* species (*P. dakotaensis*, *P. neglectus*, *P. scribneri*, and Hg51 unnamed RLN) were included as negative controls. The presence of DNA was initially confirmed by conventional PCR amplification of the internal transcribed spacer (ITS) region of ribosomal DNA (rDNA) using universal primers TW81 (forward) and AB28 (reverse) [52], followed by gel electrophoresis. To confirm species identity, PCR was performed using species-specific primers for *P. penetrans* (PP5F/PP5R), which amplify a 520 bp fragment [24]. The PCR amplification was carried out in a 25 µL reaction containing 2 µL of DNA template, 12.5 µL of 2 × DreamTaq Green PCR Master Mix (Thermo Scientific™, Waltham, MA, USA), 0.5 µM of each primer, and nuclease-free ddH_2_O. The thermal cycling conditions consisted of an initial denaturation at 95 °C for 15 min, followed by 35 cycles of denaturation at 94 °C for 30 s, annealing at 57 °C for 90 s, and extension at 72 °C for 1.5 min, with a final extension step at 72 °C for 10 min. PCR products from both primer sets, TW81/AB28 and PP5F/PP5R, were separated by gel electrophoresis on a 2% agarose gel stained with ethidium bromide (EtBr), and the banding pattern was visualized and photographed under UV light using a UVP MultiDoc-it Digital Imaging System (Analytikjena, Jena, Germany).

### 4.2. DNA Extraction from Potato Roots and Primer Selection

DNA was extracted from both infected and uninfected potato root tissues using the Fast DNA Spin Kit (MP Biomedicals, Solon, OH, USA) according to the manufacturer’s protocol. For each root sample, 0.2 g of root tissue was placed in a lysing matrix tube and homogenized using a FastPrep-24™ 5G instrument (MP Biomedicals, Solon, OH, USA) to ensure thorough cell disruption. For qPCR assay development, DNA was extracted from 0.2 g of Red Norland root tissue artificially inoculated with *P. penetrans* in the laboratory. The assay was tested using DNA extracted from infected root tissues of five different potato cultivars grown under greenhouse conditions. DNA extracted from uninfected Red Norland root tissues served as negative controls for the assay. Prior to extraction, roots were carefully separated from soil to avoid damage and washed under running tap water for 4 to 5 min to remove debris. They were then surface sterilized by dipping in 70% ethanol for 1 min, blot-dried with clean paper towels, and cut into 1–2 cm segments using surface-sterilized scissors. The root pieces were mixed thoroughly to ensure sample homogeneity.

The primer pair Pp-F (5′-GGTTTTCGGGCTCATATGGGTTC-3′) (forward) and Pp-R (5′-TTTACGCCG AGAGTGGGATTGTG-3′) (reverse) previously designed from the D2-D3 expansion region of the 28S rRNA gene of *P. penetrans*, was used in this study [22]. This primer set amplified a 111 bp fragment and was specific to *P. penetrans*, and was originally developed for its detection directly from DNA extracts of soil samples.

### 4.3. Specificity Evaluation 

Although the primer pair used in this study was previously validated by Baidoo et al. [22] for detecting and quantifying *P. penetrans* in soil, its specificity was re-evaluated using DNA extracted from potato root tissues to account for differences in sample composition and potential co-extracted organisms. Red Norland potato plants were grown in autoclaved river sand (Soil pH: 6.5, sand: 87%, silt: 6%, clay: 7%, texture: loamy sand) collected from Cass county, ND, and fresh, non-inoculated roots were harvested 30 days after planting (DAP). The roots were thoroughly washed, surface-sterilized with 70% ethanol, blot-dried, and cut into 1–2 cm pieces. To assess specificity, *P. penetrans* harvested from fresh carrot culture, four additional *Pratylenchus* species (*P. dakotaensis*, *P. neglectus*, *P. scribneri* and Hg51 unnamed RLN), and nematodes of four other genera commonly found in the fields of ND and MN were tested (Table 1). Individual nematodes were manually picked using dental picks and artificially inoculated in 0.2 g of potato roots separately. DNA was extracted from each inoculated sample, and qPCR was performed with three technical replicates per nematode species. Two negative controls, (i) DNA extracted from non-inoculated potato roots of Red Norland, and (ii) ddH_2_O (non-template control), were included in the qPCR assay, while DNA extracted from *P. penetrans* using Proteinase K method was used as a positive control.

### 4.4. Detection of qPCR Inhibitors in Potato Roots and Optimization Using BSA

The presence of qPCR inhibitors in DNA extracted from potato plant roots was assessed using a qPCR assay targeting the pGEM-T Easy vector (Promega Corp., Madison, WI, USA), following the method described by Arora and Yan [17]. Uninfected root samples from Red Norland potato cultivar, grown in autoclaved river sand on July 10, 2024, under greenhouse conditions, were collected at four different growth stages: 20 DAP (DP1), 25 DAP (DP2), 30 DAP (DP3), and 35 DAP (DP4). From each plant, roots were washed, cut into 1–2 cm pieces, mixed thoroughly, and 0.2 g of tissue was subsampled for DNA extraction (one subsample per plant). Each qPCR reaction comprised a 10 µL mix containing 1.5 µL of root DNA extract, 6.05 × 10^8^ copies of plasmid DNA template, 5.0 µL of Sso Advanced Universal SYBR Green Supermix (Bio-Rad Laboratories, Inc., Hercules, CA, USA), 0.5 µL of T7 (forward: 5′-TAATACGACTCACTATAGGG-3′), and SP6 (reverse: 5′-TATTTAGGTGACACTATAG-3′) primers (10 μM), and 1.5 µL of ddH_2_O. Each treatment was run in triplicate. Positive controls consisted of plasmid DNA and ddH_2_O without root DNA, while negative controls contained no plasmid or root DNA, only ddH_2_O. The presence of qPCR inhibitors was determined by statistically comparing the Cq values between positive controls and reactions containing root DNA using SAS software (version 9.4).

With the detection of qPCR inhibitors, four different concentrations (0.2, 0.3, 0.4, and 0.5 µg/µL) of Bovine Serum Albumin (BSA; Invitrogen, Carlsbad, CA, USA) were used to evaluate its effectiveness in neutralizing qPCR inhibitors. BSA was added to the qPCR reaction mixtures containing root DNA extracts, and qPCR was performed. The resulting Cq values were statistically compared to those of the positive control (plasmid DNA and ddH_2_O without root DNA) to determine the optimal level of BSA that reduces PCR inhibition and improves qPCR accuracy.

### 4.5. Reaction and Amplification of qPCR

The qPCR assay was carried out in a 96-well plate on a Bio-Rad CFX96 Touch Real-Time PCR Detection System (Bio-Rad) under the following cycling conditions: incubation at 95 °C for 5 min, followed by 35 cycles of 95 °C for 10 s, 66 °C for 20 s, and 72 °C for 30 s, with melting curve analysis using the default settings to assess specificity. qPCR reactions were set up with a 10 μL mix containing 1.5 μL of DNA template, 0.5 μL of each primer (10 μM), 5.0 μL of Sso Advanced Universal SYBR Green Supermix (Bio-Rad), 0.2 μL of BSA (20 μg/μL), and 2.3 μL of ddH_2_O. Data analysis was performed using Bio-Rad CFX Manager Software version 3.1, and the Cq value was determined for each reaction at default settings.

### 4.6. Sensitivity Testing

For determination of the detection sensitivity of the qPCR assay, four *P. penetrans* (mixed developmental stages, including males and females) extracted from fresh carrot disc culture were artificially inoculated in 0.2 g of fresh roots of the Red Norland potato, and DNA was extracted. The extracted DNA was diluted two-fold to create a serial dilution, and qPCR was performed on each dilution in three technical replicates. The lowest detection limit was determined as the highest dilution at which a consistent amplification signal was observed across replicates. Positive and negative controls were included as in the specificity assay.

### 4.7. Generation and Validation of Standard Curve

The qPCR standard curve was developed by inoculating Red Norland potato roots with varying densities of *P. penetrans* (1, 4, 16, 64, and 256 nematodes per 0.2 g of roots) of different life stages (juveniles and adults) obtained from fresh carrot disc cultures. DNA was extracted from artificially inoculated potato roots in three biological replicates for each density. Each sample and control had three technical replicates in the qPCR reaction. Two negative controls, (i) ddH_2_O in place of DNA template, and (ii) DNA from uninfected roots of Red Norland, and one positive control consisting of DNA of previously confirmed *P. penetrans* extracted by Proteinase K extraction were included in the qPCR assay. The standard curve was generated by plotting Cq values against the corresponding log_10_ of *P. penetrans* nematode densities used to inoculate the uninfected potato roots. The standard curve equation was obtained, and the amplification efficiency (E) was calculated using the formula, E = 10 ^(1/–m)^ − 1, where ‘*m*’ is the slope of the curve as represented in the standard curve equation.

The qPCR standard curve was validated using DNA extracts from Red Norland potato roots inoculated with *P. penetrans* at densities (3, 9, 27, 81, and 243) different from those used during initial curve development, in 0.2 g root samples. DNA was extracted in three biological replicates for each density, and qPCR assays were performed in triplicate for each sample using the same protocol as standard curve generation. The qPCR standard curve validation was evaluated by correlating the qPCR estimates of *P. penetrans* derived from the standard curve equation against the actual *P. penetrans* densities used for inoculation.

### 4.8. Greenhouse Experiments to Evaluate Reproduction of P. penetrans in Roots of Potato Cultivars

Five potato cultivars commonly grown in ND and MN, Red Norland, Caribou Russet, Modoc, Colomba, and Yukon Gold, were evaluated for their ability to support *P. penetrans* reproduction in roots in two greenhouse trials conducted under controlled conditions with 16 h of daylight and average temperature of 22 °C. Each treatment included five replications arranged in a randomized complete block design (RCBD).

In the first trial, conducted in September of 2024, a soil mixture of *P. penetrans*–infested field soil maintained under greenhouse conditions by planting the susceptible potato cultivar Red Norland and autoclaved river sand (1:3 ratio) was used for planting. Each pot was filled with 1 kg of the soil mixture. The initial nematode population in the soil was determined by extracting three subsamples using the Whitehead tray method [53] and counting under an inverted-light microscope (Zeiss Axiovert 25, Carl Zeiss Microscopy, White Plains, NY, USA), resulting in an average of 285 *P. penetrans* per kg of soil. Ten DAP, each pot was artificially inoculated with 5 mL of a suspension containing 1315 mixed-stage *P. penetrans* (juveniles and adults) extracted from fresh carrot disc cultures to supplement the existing nematode population in the soil and increase the total density to 1600 per kg of soil. Potato plants were harvested 72 DAP (December 2024), and the roots were processed for nematode extraction using the Whitehead tray method.

A second experiment was set up in January of 2025 using the remaining soil after nematodes were extracted from the first experiment; the soil was mixed with autoclaved river sand in a 4:1 ratio. The initial *P. penetrans* density, determined by the Whitehead tray method, was 1440 nematodes per kg of soil. The same five cultivars were planted and harvested 72 DAP (April, 2025), according to the same procedures as the first trial.

### 4.9. Assessment of P. penetrans Reproduction in Roots of Potato Cultivars

After harvesting, roots were carefully separated from the soil. Roots were washed under running water to remove residual soil, cut into 1–2 cm pieces using sterile scissors, mixed thoroughly, and homogenized. From each homogenized root sample, 1 g was kept for DNA extraction to assess the qPCR assay (described separately). The remaining root material was used for nematode extraction using the Whitehead tray method. In the first trial, the remaining root tissue was divided into three equal portions and processed separately using the Whitehead tray extraction method. In the second trial, the entire remaining root sample was processed at once. The final *P. penetrans* population in roots was extrapolated based on the total root (in grams) of the potato cultivars observed in both the first and second trials.

### 4.10. Detection and Quantification of P. penetrans from Potato Plant Roots Using qPCR Assay

From each homogenized root sample, one gram was processed for DNA extraction as previously described. For DNA extraction, one gram of roots was treated with 70% ethanol for 1 min, blot-dried with clean paper towels, and divided into three subsamples. DNA was extracted from 0.2 g of each subsample, and the qPCR assay was performed in triplicate. Two DNA extracts used for developing the standard curve equation (1 and 256 *P. penetrans*/0.2 g of roots) were used as positive controls. Two negative controls containing (i) ddH_2_O in place of DNA template, and (ii) root DNA from non-inoculated potato plants, were also included in this qPCR assay. The nematode population in 0.2 g of root tissue was estimated using the standard curve equation (y = −3.304x + 29.961) and extrapolated to one gram of root tissue. For Whitehead tray extractions, nematode suspensions were collected after 48 h, counted under an inverted-light microscope, then converted to one-gram root equivalent for each of the five potato cultivars. Correlation analysis was conducted using SAS software (version 9.4) to compare nematode estimates obtained by qPCR with those from the Whitehead tray extractions followed by microscopic counting.

### 4.11. Statistical Analysis

All statistical analyses were conducted using SAS software (version 9.4). The PROC GLM procedure was used to assess the effects of root DNA extracts from samples collected at different DAP on qPCR inhibition, and the effect of different BSA concentrations on inhibitor neutralization based on the Cq values. Tukey’s honestly significant difference (HSD) test was used for mean separation to identify significant differences in Cq values (*p* < 0.05), with treatments compared to the positive control. The PROC GLIMMIX procedure was used to analyze the mean final population density in roots (in grams) among five potato cultivars at a 5% significance level, followed by Tukey’s HSD test to determine significant differences between cultivar means. *Pratylenchus penetrans* populations determined by microscopic counts and qPCR assay in the five potato cultivars were compared using pairwise *t*-tests (PROC TTEST) (*p* < 0.05). The PROC REG procedure was used to evaluate the correlation (*p* < 0.05) between nematode counts obtained by qPCR assay and those determined using the microscopic method. All graphs were generated using Microsoft Excel (version 2019, Microsoft Corporation, Redmond, WA, USA).

## 5. Conclusions

RLNs are difficult to manage due to their wide host range and ability to survive across diverse cropping systems. Their endo-parasitic lifestyle and presence within root tissues often result in infestations going undetected until substantial damage has occurred. In this study, we developed a SYBR Green-based qPCR assay for the direct detection and quantification of *P. penetrans* in potato roots, the nematode’s primary habitat, without the need for prior nematode extraction. The assay was optimized for direct use with root DNA extracts, enabling in planta detection and quantification with high sensitivity and specificity. The strong linear correlation of qPCR estimates with microscopic nematode counts across multiple potato cultivars confirmed its accuracy and robustness. By eliminating the need for nematode extraction, this method provides a rapid, and user-friendly alternative to traditional diagnostic techniques. This molecular tool holds significant potential for diagnostic laboratories and research engaged in routine monitoring, surveillance, and epidemiological studies. It may also facilitate investigations into temporal population dynamics, spatial distribution, and host–nematode interactions under various environmental conditions.

## Figures and Tables

**Table 1:** Plant-parasitic nematode species used to evaluate the specificity of primers for detecting *Pratylenchus penetrans* in the qPCR assay.

Nematode Species ^p^	Cq ^q^	Origin	Reference
*Pratylenchus penetrans*	29.92 ± 0.06	MN, USA	This study
*P. penetrans*	31.21 ± 0.22	MN, USA	This study
*P. dakotaensis*	N/A	ND, USA	[31]
*P. neglectus*	N/A	ND, USA	[32]
*P. scribneri*	N/A	ND, USA	[17]
Hg51 unnamed root-lesion nematode	N/A	ND, USA	[33]
*Helicotylenchus microlobus*	N/A	ND, USA	Author’s collection
*Heterodera glycines*	N/A	ND, USA	Author’s collection
*Paratrichodorus allius*	N/A	ND, USA	Author’s collection
*Paratylenchus nanus*	N/A	ND, USA	Author’s collection
Positive control 1 ^r^	27.41 ± 0.12	MN, USA	This study
Positive control 2 ^s^	23.43 ± 0.17	MN, USA	This study
Negative control 1 ^t^	N/A		
Negative control 2 ^u^	N/A		

^p^ Nematode species inoculated into 0.2 g of uninfected ‘Red Norland’ potato roots prior to DNA extraction (except for positive and negative controls), to evaluate the specificity of the qPCR assay using root DNA extracts. ^q^ Quantification cycle (Cq) values represented as the mean ± standard deviation of technical replicates (*n* = 3). N/A indicates no amplification. ^r^ Positive control 1 indicates DNA extracted by Proteinase K method with one *P. penetrans* was used as DNA template. ^s^ Positive control 2 indicates that DNA extracted by Proteinase K method with four *P. penetrans* was used as DNA template. ^t^ Negative control 1 indicates non-template control: ddH_2_O in place of DNA template. ^u^ Negative control 2 indicates DNA from uninfected roots of ‘Red Norland’ was used as the DNA template.

**Table 2:** Quantification cycle (Cq) values of root DNA extracts collected at different days after planting (DAP) to assess the presence of qPCR inhibitors.

Treatments	DAP (Days) ^p^	Cq ^q^
DP1	20	19.32 ± 0.11 ^b^
DP2	25	21.09 ± 0.15 ^a^
DP3	30	19.15 ± 0.13 ^b^
DP4	35	19.19 ± 0.09 ^b^
Positive control ^r^		16.92 ± 0.10 ^c^
Negative control ^s^		N/A
Pr > F ^t^		<0.0001

^p^ DAP represents the days after planting at which the root samples were taken for DNA extraction for assessment of qPCR inhibitors from uninfected ‘Red Norland’ potato planted in autoclaved river sand under greenhouse conditions on 10 July 2024. ^q^ Quantification cycle (Cq) values represented as the mean ± standard deviation of the technical replicates (*n* = 3). Values followed by same letters are not significantly different (Tukey’s HSD, *p* < 0.05) (^a–c^). N/A indicates no amplification in quantitative PCR. ^r^ Positive control consisting of plasmid DNA and ddH_2_O without root DNA. ^s^ Negative controls containing only ddH_2_O with no plasmid or root DNA. ^t^ Pr > F is Probability > F value.

**Table 3:** Effect of bovine serum albumin (BSA) concentrations in neutralizing the PCR inhibitors present in root DNA extracts based on quantification cycle (Cq) values.

BSA Concentration (µg/µL)	Cq ^p^
0	19.69 ± 0.85 ^a^
0.2	18.52 ± 0.57 ^b^
0.3	18.43 ± 0.52 ^b,c^
0.4	17.72 ± 0.48 ^d^
0.5	17.78 ± 0.33 ^c,d^
Positive control ^q^	16.92 ± 0.10 ^d^
Pr > F ^r^	<0.0001

^p^ Quantification cycle (Cq) values represented as the mean ± standard deviation (*n* = 12) of four root DNA extract samples taken at 20, 25, 30 and 35 days after planting (DAP) from uninfected ‘Red Norland’ potato grown under greenhouse conditions, each ran in three technical replicates in the qPCR assay. Potato plants were planted in autoclaved river sand on 10 July 2024. Values followed by the same letter(s) are not significantly different (Tukey’s HSD, *p* < 0.05) (^a–d^). ^q^ Positive control consisting of plasmid DNA and ddH_2_O without root DNA. ^r^ Pr > F is Probability > F value.

**Table 4:** Quantification cycle (Cq) values for serial dilution of DNA extract of ‘Red Norland’ potato roots artificially inoculated with four *P. penetrans* for analysis of detection sensitivity of the qPCR assay.

Serial Dilution	*P. penetrans* Equivalents/0.2 g of Roots	Cq ^p^
1	4	27.72 ± 0.14
1:2	2	27.95 ± 0.12
1:4	1	28.31 ± 0.07
1:8	5 × 10^−1^	29.43 ± 0.10
1:16	2.5 × 10^−1^	30.51 ± 0.09
1:32	1.25 × 10^−1^	31.75 ± 0.11
1:64	6.25 × 10^−2^	32.88 ± 0.12
1:128	3.13 × 10^−2^	33.85 ± 0.07
1:256	1.56 × 10^−2^	34.77 ± 0.07
1:512	7.81 × 10^−3^	N/A
1:1024	3.91 × 10^−3^	N/A
Negative control 1 ^q^		N/A
Negative control 2 ^r^		N/A
Positive control 1 ^s^		27.30 ± 0.07
Positive control 2 ^t^		23.38 ± 0.08
Coefficient (*R*^2^)		0.98
Efficiency (E)		106.47%

^p^ Quantification cycle (Cq) values represented as the mean ± standard deviation of technical replicates (*n* = 3). N/A indicates no amplification in quantitative PCR. ^q^ Negative control 1 indicates non-template control: ddH_2_O in place of DNA template. ^r^ Negative control 2 indicates that DNA from uninfected roots of ‘Red Norland’ was used as a DNA template. ^s^ Positive control 1 indicates DNA extracted by Proteinase K method with one *P. penetrans* was used as the DNA template. ^t^ Positive control 2 indicates DNA extracted by Proteinase K method with four *P. penetrans* was used as the DNA template.

**Table 5:** Mean final population density of *Pratylenchus penetrans* in roots per plant of potato cultivars.

Potato Cultivars	Final Population Density in Roots Per Plant ^p^					
	Trial 1			Trial 2		
	Root Weight (g) ^q^	Microscopic Counts ^r^	qPCR Estimates ^s^	Root Weight (g)	Microscopic Counts	qPCR Estimates
Caribou Russet	13.52	4728 ± 773 ^a^	4558 ± 942 ^a,b^	12.80	3318 ± 412 ^b^	4091 ± 1381 ^a^
Colomba	13.30	2101 ± 262 ^c^	2166 ± 372 ^c^	11.72	1880 ± 291 ^c^	2497 ± 609 *^,b,c^
Modoc	13.64	1952 ± 175 ^c^	2276 ± 199 ^c^	10.96	1709 ± 187 ^c^	2087 ± 294 **^,c^
Red Norland	13.94	5420 ± 403 ^a^	5473 ± 634 ^a^	13.78	4023 ± 411 ^a^	5141 ± 731 *^,a^
Yukon Gold	14.12	3712 ± 342 ^b^	3863 ± 707 ^b^	12.22	3287 ± 370 ^b^	3725 ± 505 **^,a,b^
Pr > F ^t^		<0.0001	<0.0001		<0.0001	<0.0001

^p^ Final population density of *P. penetrans* in roots per plant for potato cultivars in Trial 1 and Trial 2. Trial 1 was planted in September 2024 and harvested in December 2024 (72 days after planting (DAP)) with an initial nematode density of 1500 *P. penetrans* per kg of soil. Trial 2 was conducted from January to April 2025 with an initial nematode density of 1440 P. *penetrans* per kg of soil. ^q^ Root weight was the average root weight (in grams) for potato cultivars across replicates (*n* = 5 for both trials). ^r^ Microscopic counts were the final population densities of *P. penetrans* in roots determined by Whitehead tray extraction followed by microscopic counting. For each of the five replicates in the greenhouse, the roots that remained after 1 g was removed for DNA extraction were used for nematode extraction. Values are the mean ± standard deviation (*n* = 15 for Trial 1 and *n* = 5 for Trial 2), extrapolated to the total root weight for each cultivar. In Trial 1, three independent nematode extractions were performed per greenhouse replicate; in Trial 2, a single extraction was performed per greenhouse replicate. ^s^ qPCR estimates were the final population densities of *P. penetrans* in the roots, as estimated by the qPCR assay. For each of the five greenhouse replicates, 1 g of root tissue was sampled from the total root mass and used for DNA extraction. Values are the mean ± standard deviation (*n*= 45 for both trials), extrapolated to the total root weight for each cultivar. For each greenhouse replicate, three independent DNA extractions were performed, and each DNA sample was analyzed in three technical qPCR replicates. Asterisks indicate significant differences between *P. penetrans* populations determined by microscopic counts and qPCR assay within each trial: *p* < 0.05 (*) and *p* < 0.01 (**). Within each column, values followed by the same letter(s) are not significantly different (Tukey’s HSD, *p* < 0.05) (^a–c^). ^t^ Pr > F is the Probability > F value.

## Data Availability

The data generated or analyzed during this study are included in this article.

## Supplementary Materials

The following supporting information can be downloaded at https://www.mdpi.com/article/10.3390/ijms26167711/s1.
