# Supplementary material for: Development of a Real-Time Quantitative PCR Assay for Direct Detection and Quantification of the Root-Lesion Nematode Pratylenchus penetrans in Potato Roots
Source: Int J Mol Sci. 2025 Aug 9;26(16):7711. doi: 10.3390/ijms26167711 (PMC12386274; doi:10.3390/ijms26167711)
Supplement: Supplementary file 1 [file ijms-26-07711-s001.zip › ijms-3761313-supplementary.pdf]

International Journal of
Molecular Sciences

MDPI

Supplementary materials

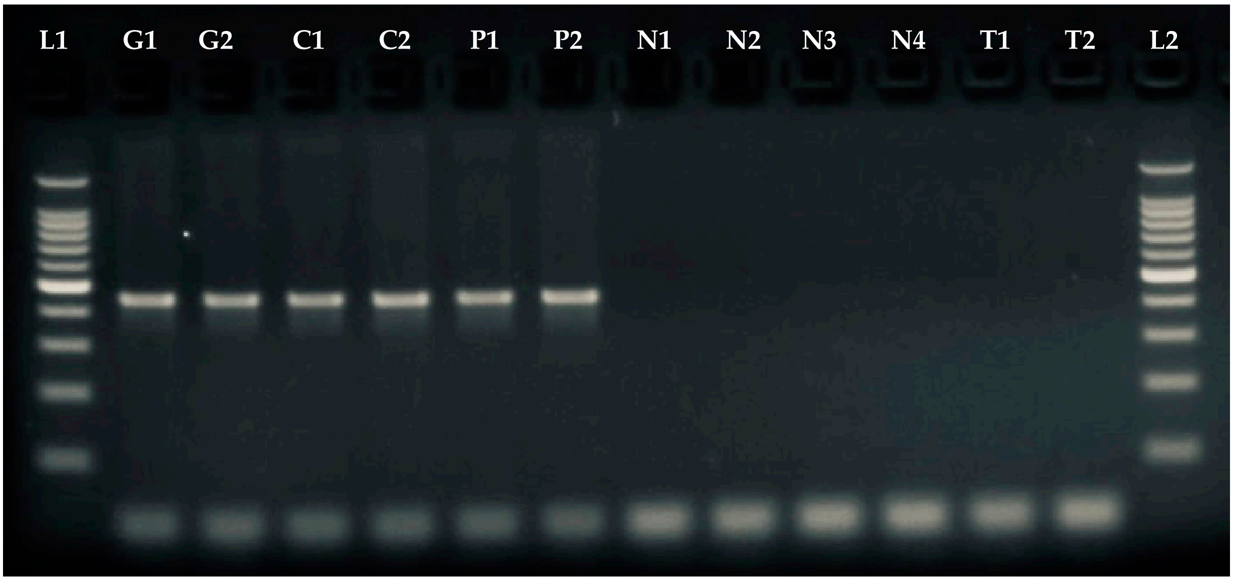

Supplementary Figure S1. Gel image showing PCR amplification using the species-specific primer pair PP5F/PP5R for *Pratylenchus penetrans*. Lanes L1 and L2 represent a 100 bp DNA ladder. Lanes G1 and G2 contain DNA from single root-lesion nematode (RLN) individuals extracted from greenhouse-grown susceptible cultivar 'Red Norland'. Lanes C1 and C2 show DNA from single RLN individuals extracted from carrot disc cultures. Lanes P1 and P2 represent positive controls with previously confirmed *P. penetrans* DNA. Lanes N1 through N4 are negative controls: N1 contains *P. scribneri* DNA, N2 contains *P. neglectus* DNA, N3 contains *P. dakotaensis* DNA, and N4 contains DNA from an Hg51 unnamed RLN species. Lanes T1 and T2 are non-template controls using nuclease-free water (ddH$_2$O).
